# Beyond the Kidneys: Multisystem Systemic Lupus Erythematosus Presenting With Cardiac Tamponade, Class V Lupus Nephritis, Dysphagia, and Peripheral Neurologic Manifestations

**DOI:** 10.7759/cureus.111882

**Published:** 2026-07-01

**Authors:** Nickolle A Cruz Figueroa, Gonzalo J Martinez-Ruiz, Bak Nin Choi Reina, Sebastian S Leon-Laussel, Gladymar Gonzalez Marrero

**Affiliations:** 1 Internal Medicine, Central Caribbean University, Bayamon, PRI

**Keywords:** cardiac tamponade, lupus nephritis, membranous glomeluropathy, rheumatology care, systemic lupus erythromatosus

## Abstract

Systemic lupus erythematosus (SLE) is a chronic autoimmune disorder characterized by immune dysregulation, immune complex deposition, and multisystem inflammation with highly variable clinical manifestations. Although lupus nephritis and serositis are common complications, the simultaneous occurrence of biopsy-proven membranous lupus nephritis, cardiac tamponade, polyserositis, dysphagia, and peripheral neurologic manifestations is uncommon and presents significant diagnostic and therapeutic challenges.

We present the case of a 37-year-old Hispanic woman with newly diagnosed SLE who developed progressive dysphagia, peripheral sensory deficits, pleural and pericardial involvement complicated by cardiac tamponade, ascites, and biopsy-proven International Society of Nephrology/Renal Pathology Society (ISN/RPS) Class V lupus nephritis. Laboratory evaluation demonstrated a homogeneous antinuclear antibody titer greater than 1:2560, elevated anti-double-stranded DNA antibodies, hypocomplementemia, and approximately 2 g/day of proteinuria. Cross-sectional imaging demonstrated extensive serosal involvement, including bilateral pleural effusions, a large pericardial effusion, pelvic ascites, and perihepatic free fluid. Renal biopsy demonstrated ISN/RPS Class V membranous lupus nephritis with subepithelial, intramembranous, and mesangial immune-type electron-dense deposits and near-global podocyte foot process effacement on electron microscopy. The patient required urgent pericardiocentesis and multidisciplinary management with pulse corticosteroids, hydroxychloroquine, mycophenolate mofetil, and belimumab. Following treatment, her clinical condition improved with resolution of cardiac tamponade and stabilization of her multisystem disease.

This case highlights the protean nature of SLE and emphasizes the importance of recognizing simultaneous renal, cardiac, neurologic, gastrointestinal, and serosal involvement. Early multidisciplinary evaluation and prompt immunosuppressive therapy are essential to preventing irreversible organ damage and improving clinical outcomes.

## Introduction

Systemic lupus erythematosus (SLE) is a chronic autoimmune disease characterized by loss of immune tolerance, autoantibody production, immune complex deposition, and complement activation, leading to inflammation and tissue injury involving virtually any organ system [1]. The disease predominantly affects women of childbearing age and exhibits a highly variable clinical course ranging from mild mucocutaneous manifestations to severe multiorgan dysfunction.

Renal involvement develops in nearly half of patients with SLE and remains one of the major determinants of long-term morbidity and mortality. Lupus nephritis comprises a spectrum of histopathologic patterns classified by the International Society of Nephrology/Renal Pathology Society (ISN/RPS), with Class V membranous lupus nephritis accounting for approximately 10-20% of cases. Membranous lupus nephritis is characterized by subepithelial immune complex deposition and frequently presents with significant proteinuria while preserving glomerular architecture [2].

Serosal involvement is another common manifestation of active SLE and may affect the pleura, pericardium, or peritoneum. Pericarditis represents the most frequent cardiac manifestation of the disease; however, progression to clinically significant cardiac tamponade is uncommon and may indicate severe systemic inflammation requiring urgent intervention [3]. Simultaneous involvement of multiple serosal surfaces resulting in polyserositis is encountered less frequently and often reflects heightened disease activity.

Neurologic and gastrointestinal manifestations of SLE are diverse and frequently underrecognized. Peripheral sensory deficits, autonomic dysfunction, and dysphagia may occur through inflammatory, vascular, or immune-mediated mechanisms and can contribute to delayed diagnosis and increased morbidity [4].

We present a case of newly diagnosed SLE with simultaneous biopsy-proven ISN/RPS Class V lupus nephritis, extensive polyserositis, cardiac tamponade requiring urgent pericardiocentesis, progressive dysphagia, and peripheral neurological manifestations. The coexistence of these findings within a single disease flare highlights the protean nature of SLE and underscores the importance of early multidisciplinary recognition and management.

## Case presentation

A 37-year-old Hispanic woman with no known history of autoimmune disease presented with progressive multisystem complaints that had developed over several weeks. She reported generalized fatigue, polyarthralgias involving multiple joints, progressive hair loss, photosensitivity, Raynaud phenomenon, bilateral lower extremity edema, and worsening shortness of breath associated with intermittent chest discomfort. She also described progressive dysphagia involving both solids and liquids and diffuse, symmetric diminished sensation affecting all extremities, with impaired perception of light touch, pain, and temperature, raising concern for gastrointestinal and peripheral neurological involvement.

Initial laboratory evaluation demonstrated significant autoimmune activity. Antinuclear antibody testing was positive with a homogeneous pattern at a titer greater than 1:2560. Anti-double-stranded DNA antibodies were markedly elevated at 180 IU/mL, while complement studies demonstrated decreased C3 and C4 levels, consistent with active immune complex-mediated disease activity [1]. Urinalysis revealed significant proteinuria, and a 24-hour urine collection quantified protein excretion at approximately 2.0 g/day. Given the combination of active serologic markers and renal involvement, lupus nephritis was suspected, and nephrology was consulted for further evaluation.

**Figure 1:**
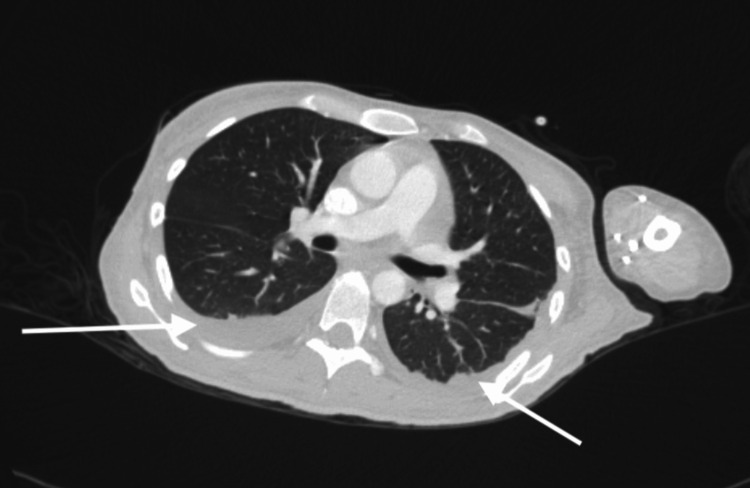
Bilateral pleural effusions.

**Figure 2:**
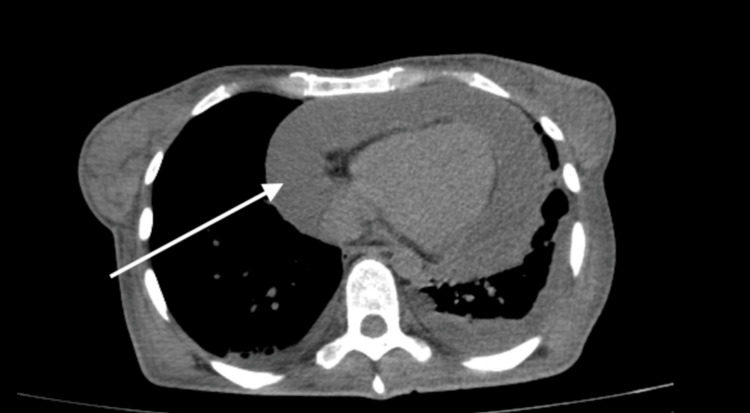
Pericardial effusion.

**Figure 3:**
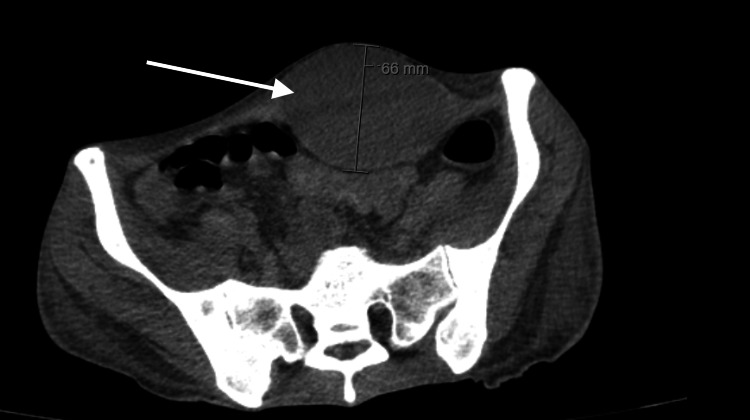
Large left rectus abdominis intramuscular abscess.

**Figure 4:**
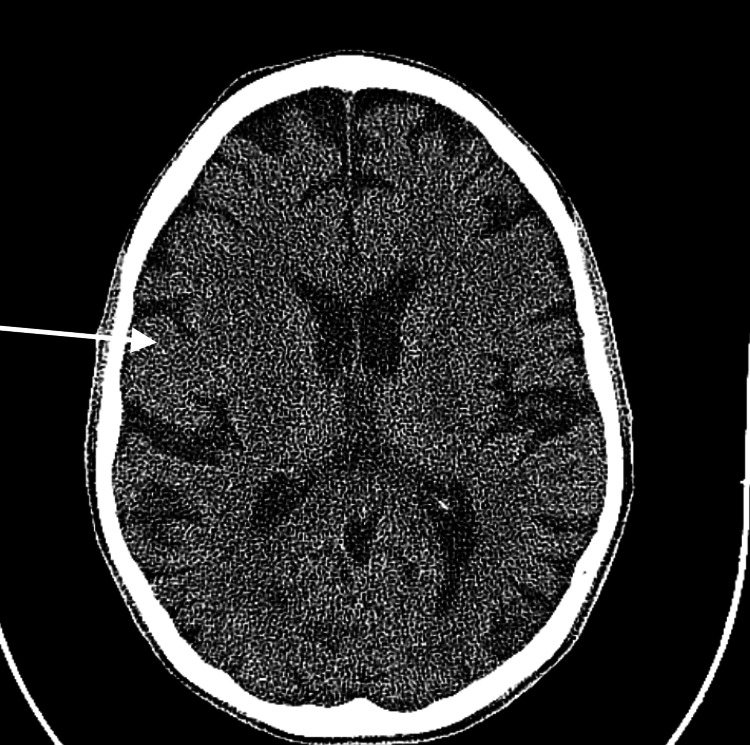
Acute subarachnoid hemorrhage.

Computed tomography (CT) of the chest demonstrated a moderate right pleural effusion with associated compressive atelectatic changes and a moderate left pleural effusion, findings compatible with active lupus pleuritis and serositis (Figure 1). A circumferential pericardial effusion was also identified (Figure 2). CT of the abdomen and pelvis demonstrated mild pelvic ascites and a large left rectus abdominis intramuscular fluid collection favored to represent an abscess measuring approximately 66 mm in maximal depth together with mild perihepatic free fluid, supporting diffuse serosal involvement and polyserositis (Figure 3). CT of the chest demonstrated a moderate right pleural effusion with associated compressive atelectatic changes and a moderate left pleural effusion, findings compatible with active lupus pleuritis and serositis (Figure 1). A circumferential pericardial effusion was also identified (Figure 2). CT of the abdomen and pelvis demonstrated mild pelvic ascites and a large left rectus abdominis intramuscular fluid collection favored to represent an abscess measuring approximately 66 mm in maximal depth, together with mild perihepatic free fluid, supporting diffuse serosal involvement and polyserositis (Figure 3). A CT of the head demonstrated an acute subarachnoid hemorrhage (Figure 4), raising concern for a ruptured intracranial aneurysm. In the setting of suspected Libman-Sacks endocarditis, embolic phenomena and aneurysmal vascular complications were considered, prompting further evaluation with magnetic resonance angiography (MRA) of the brain and echocardiography. MRA demonstrated no evidence of intracranial aneurysm, large-vessel occlusion, or high-flow vascular malformation.

During hospitalization, the patient's respiratory symptoms progressively worsened with increasing dyspnea and chest pain. Transthoracic echocardiography demonstrated preserved left ventricular systolic function with a significant circumferential pericardial effusion. Clinical and echocardiographic findings became concerning for cardiac tamponade physiology. Emergent pericardiocentesis was performed because of cardiac tamponade. Removal of approximately 400 mL of pericardial fluid, resulting in significant symptomatic improvement. Cytologic examination of the pericardial fluid was negative for malignancy and demonstrated an acute inflammatory process characterized by abundant neutrophils, few mesothelial cells, and cellular debris, supporting an inflammatory etiology consistent with lupus serositis. The coexistence of pleural effusions, pericardial effusion, ascites, and perihepatic fluid was consistent with extensive lupus-associated polyserositis and reflected severe systemic disease activity [3].

**Figure 5:**
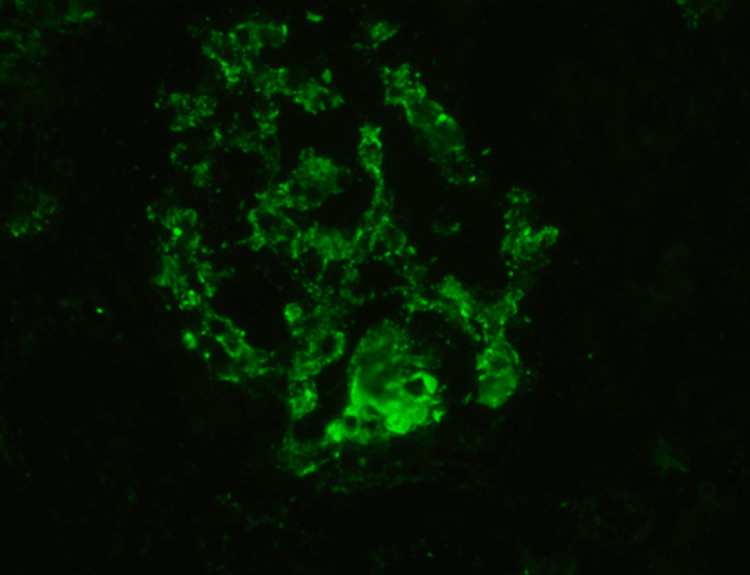
Trace granular staining in capillary loops for IgG. Examination of submitted tissue reveals renal cortex, containing up to 19 glomeruli per level section. There is trace granular staining in capillary loops for IgG, IgM, C3, C1q, kappa, and lambda. There is no significant glomerular staining for IgA, albumin, or fibrinogen. Kappa and lambda highlight the tubular casts about equally. There is no significant staining in tubular basement membranes or vessel walls. All polyclonal antibodies used for immunofluorescence staining have been previously tested and shown to have appropriate reactivities with positive control specimens. Negative control is adequate.

**Figure 6:**
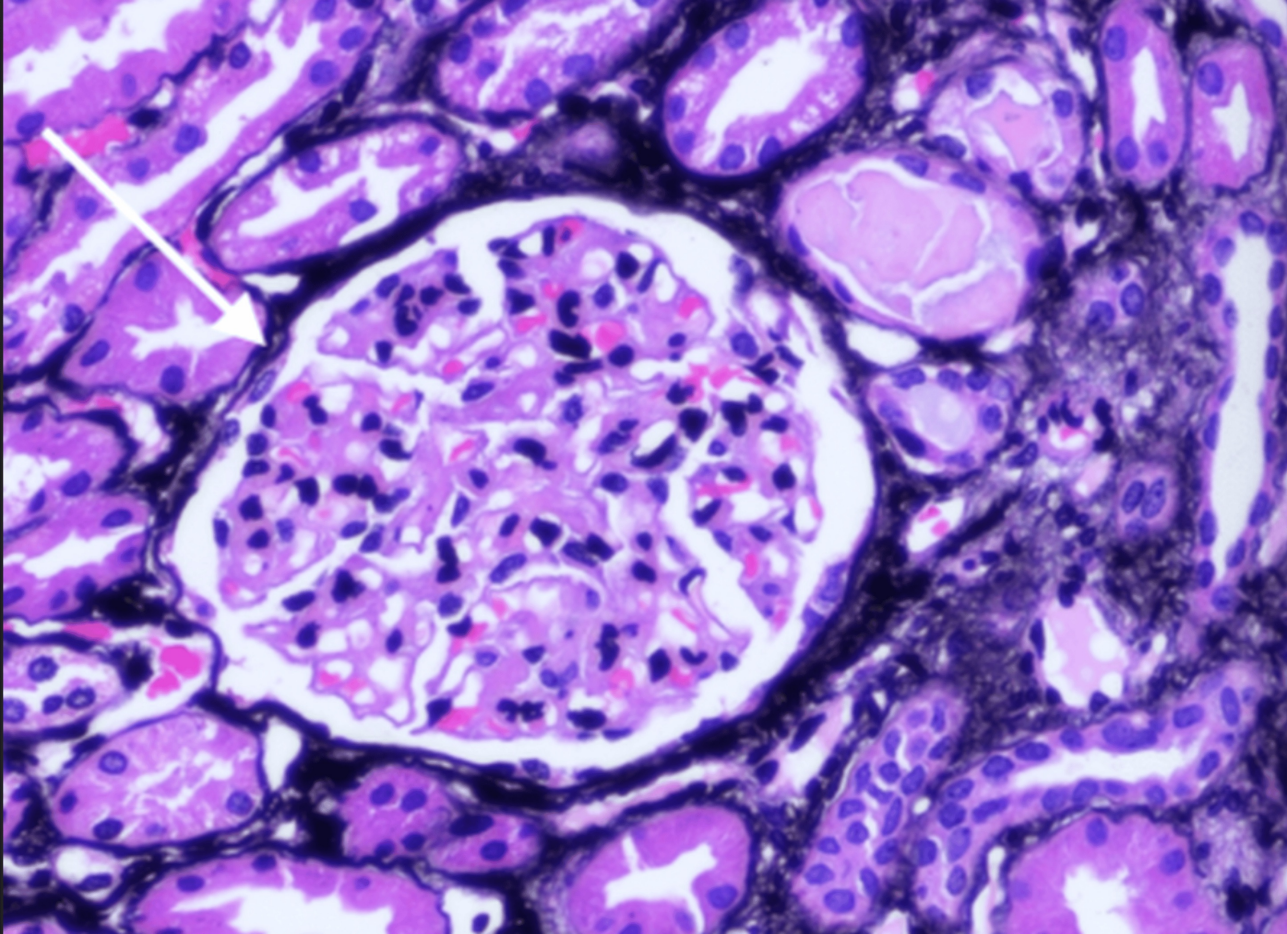
Capillary walls with minimal thickening. Up to 37 glomeruli are present for light microscopic evaluation, of which none are globally sclerosed. The glomeruli are not significantly enlarged. There is no segmental fibrinoid necrosis, endocapillary proliferation, or crescents. The mesangium shows no significant increase in matrix deposition or cellularity. The capillary loops show minimal thickening with spikes on silver methenamine stain. Tubules and interstitium: There is mild interstitial fibrosis associated with tubular atrophy, comprising 5-10% of the biopsy specimen. There is no significant interstitial infiltrate present. Proteinaceous casts are present in the tubules, some fractured, with cellular reaction. Vessels: The artery and arterioles show no abnormalities. There is no overt vasculitis or vascular necrosis. Special stains: The capillary loops show minimal thickening. Masson trichrome, silver methenamine, and PAS stains were necessary for evaluation of this biopsy. Masson trichrome, silver methenamine, and PAS stains showed normal staining patterns of internal control tissue matrix structures.

Because of persistent proteinuria and active serologic disease, a percutaneous renal biopsy was performed. Light microscopy demonstrated preserved glomerular architecture without significant proliferative lesions or crescent formation. Immunofluorescence demonstrated near "full-house" capillary loop staining with positivity for IgG, IgM, C3, and C1q but without significant IgA staining (Figure 5). The biopsy also demonstrated minimal glomerular capillary wall thickening with spikes on silver methenamine stain, consistent with membranous lupus nephritis (Figure 6).

Mild interstitial fibrosis and tubular atrophy involving approximately 5-10% of the sampled cortex were present. The overall histopathologic findings established the diagnosis of the International Society of Nephrology/Renal Pathology Society Class V membranous lupus nephritis [2].

Progressive dysphagia involving both solids and liquids persisted throughout the hospitalization. Simultaneously, the patient continued to report diffuse, symmetric diminished sensation involving all extremities, with impaired perception of light touch, pain, and temperature. Although CT of the head demonstrated an acute subarachnoid hemorrhage, these symptoms were not explained by the imaging findings and were considered compatible with neurologic and gastrointestinal complications of active SLE, reflecting the multisystem nature of the disease process [4].

The patient fulfilled multiple clinical and immunologic criteria for SLE, including positive antinuclear antibodies, elevated anti-double-stranded DNA antibodies, hypocomplementemia, alopecia, inflammatory arthralgias, Raynaud phenomenon, serositis, biopsy-proven lupus nephritis, and multisystem organ involvement.

Given the extensive disease burden, a multidisciplinary team consisting of rheumatology, nephrology, cardiology, internal medicine, and additional consulting services was assembled. Rheumatology recommended aggressive immunosuppressive therapy with pulse intravenous methylprednisolone followed by high-dose corticosteroids, hydroxychloroquine, mycophenolate mofetil, and weekly belimumab. Additional autoimmune serologies, including antiphospholipid antibodies and extractable nuclear antigen testing, were obtained, and appropriate prophylactic measures for thromboembolic and infectious complications were initiated.

Following pericardiocentesis and initiation of immunosuppressive therapy, the patient's respiratory symptoms gradually improved, and her systemic manifestations stabilized. The combination of biopsy-proven Class V lupus nephritis, extensive polyserositis with cardiac tamponade, dysphagia, and peripheral sensory abnormalities highlights the importance of maintaining a broad differential diagnosis and pursuing comprehensive organ-specific evaluation in patients presenting with active lupus.

## Discussion

SLE is a chronic autoimmune disease characterized by immune dysregulation, autoantibody production, and immune complex deposition, resulting in inflammation that may affect virtually any organ system [1]. Although isolated manifestations such as arthritis, nephritis, or serositis are common, simultaneous involvement of multiple major organ systems represents a high disease activity state and may create significant diagnostic and therapeutic challenges. Our patient presented with concurrent lupus nephritis, polyserositis, cardiac tamponade, dysphagia, and peripheral neurologic manifestations, illustrating the protean nature of active SLE.

Renal involvement remains one of the most important predictors of long-term morbidity and mortality in SLE. Lupus nephritis develops in a substantial proportion of patients and demonstrates a wide spectrum of histopathologic patterns. Class V membranous lupus nephritis is characterized by immune complex deposition along the glomerular basement membrane and frequently presents with significant proteinuria while maintaining relatively preserved glomerular architecture [2]. In our patient, active serologic disease, together with proteinuria, prompted renal biopsy, which confirmed ISN/RPS Class V lupus nephritis. This case highlights the importance of tissue diagnosis, as clinical findings alone cannot reliably predict the underlying histopathologic subtype or guide optimal therapy.

Another remarkable feature of this case was the extensive serosal involvement. Serositis is a well-recognized manifestation of SLE and may involve the pleura, pericardium, and peritoneum. Although pericardial involvement is relatively common, progression to cardiac tamponade remains an uncommon but potentially life-threatening complication requiring prompt recognition and intervention [3]. Our patient developed extensive polyserositis involving multiple serosal surfaces, ultimately requiring urgent pericardiocentesis because of hemodynamic compromise. The coexistence of pleural effusions, pericardial effusion, and ascites reflected severe systemic inflammatory activity and emphasized the need for comprehensive evaluation of patients presenting with active lupus flares. The diagnosis of lupus pericarditis was further supported by cytologic analysis of the pericardial fluid, which was negative for malignancy and demonstrated an acute inflammatory process consistent with inflammatory serositis.

The patient's progressive dysphagia and peripheral sensory deficits further demonstrated the multisystem nature of the disease. Neurologic involvement in SLE encompasses a broad spectrum of central and peripheral manifestations resulting from inflammatory, vascular, and autoimmune mechanisms [4]. Similarly, gastrointestinal symptoms may arise secondary to smooth muscle dysfunction, autonomic dysfunction, vasculitis, or associated neuromuscular involvement. Although these manifestations are less frequently recognized than renal or musculoskeletal disease, they may significantly affect quality of life and contribute to delays in diagnosis. In this patient, the coexistence of dysphagia and peripheral sensory abnormalities in the setting of active systemic disease suggested diffuse immune-mediated involvement extending beyond the traditionally recognized organ systems. The patient's acute subarachnoid hemorrhage further broadened the differential diagnosis because SLE may be associated with cerebral vasculitis, thromboembolic events, and, less commonly, intracranial aneurysms or hemorrhagic complications. In this case, subsequent MRA demonstrated no intracranial aneurysm, large-vessel occlusion, or high-flow vascular malformation, making an aneurysmal source less likely. The neurologic findings were therefore managed conservatively while immunosuppressive therapy was directed toward controlling the underlying SLE. The patient's hospitalization was further complicated by a secondary intramuscular abscess due to carbapenemase-producing Enterobacter cloacae, which required image-guided drainage and targeted antimicrobial therapy with cefiderocol. This infectious complication was managed separately from the underlying autoimmune disease and highlights the importance of maintaining vigilance for concomitant infections in immunocompromised patients with SLE.

The management of severe multisystem SLE requires close collaboration among multiple specialties. In this case, rheumatology, nephrology, cardiology, and internal medicine worked together to establish the diagnosis, address life-threatening complications, and initiate disease-modifying therapy. Current management strategies emphasize rapid control of systemic inflammation while minimizing cumulative organ damage and long-term treatment toxicity [1,2]. Our patient was treated with pulse corticosteroids followed by high-dose glucocorticoids, hydroxychloroquine, mycophenolate mofetil, and belimumab in addition to urgent pericardiocentesis for cardiac tamponade. The favorable clinical response following initiation of therapy underscores the importance of early recognition and prompt multidisciplinary intervention.

This case expands on prior literature showing that SLE can present as a true multisystem disease, with serosal, renal, gastrointestinal, and neurologic involvement occurring simultaneously. Pericardial effusion is common in SLE, but progression to cardiac tamponade remains rare and is usually reported as an unusual initial or severe manifestation of active disease [5]. Class V lupus nephritis represents membranous lupus nephritis and is strongly associated with proteinuria, requiring kidney-directed therapy guided by Kidney Disease: Improving Global Outcomes (KDIGO) recommendations [6]. Dysphagia in SLE may reflect esophageal dysmotility, reflux, medication effects, overlap with Sjögren syndrome, or inflammatory/autoimmune neuromuscular involvement [7,8]. Peripheral neurological manifestations, although less common than central nervous system disease, are recognized within the neuropsychiatric spectrum of SLE and may include polyneuropathy, mononeuropathy, cranial neuropathy, and small-fiber neuropathy [9,10].

## Conclusions

SLE is a highly heterogeneous autoimmune disease capable of producing simultaneous multiorgan involvement with potentially life-threatening complications. This case demonstrates an uncommon presentation of newly diagnosed SLE characterized by biopsy-proven ISN/RPS Class V lupus nephritis, extensive polyserositis complicated by cardiac tamponade, and suspected gastrointestinal and peripheral neurologic manifestations occurring during a single disease flare.

The coexistence of confirmed renal and cardiac involvement with suspected gastrointestinal and neurologic manifestations highlights the importance of maintaining a broad differential diagnosis when evaluating patients with active SLE, particularly when new symptoms cannot be explained by a single organ system. Early recognition of multisystem disease, timely diagnostic evaluation, including tissue biopsy when appropriate, and prompt multidisciplinary management are essential to preventing irreversible organ damage. This case further emphasizes the value of collaboration among rheumatology, nephrology, cardiology, neurology, and internal medicine in the management of complex SLE presentations. Recognition of atypical manifestations and early initiation of contemporary immunosuppressive therapy may improve both short- and long-term outcomes in patients with severe systemic disease.
